# A Silk Fibroin Bio-Transient Solution Processable Memristor

**DOI:** 10.1038/s41598-017-15395-5

**Published:** 2017-11-07

**Authors:** Jason Yong, Basem Hassan, You Liang, Kumaravelu Ganesan, Ranjith Rajasekharan, Robin Evans, Gary Egan, Omid Kavehei, Jingliang Li, Gursharan Chana, Babak Nasr, Efstratios Skafidas

**Affiliations:** 1 Centre for Neural Engineering, The University of Melbourne, Carlton, VIC 3053 Australia; 2 Department of Electrical and Electronic Engineering, University of Melbourne, Parkville, VIC 3010 Australia; 3 School of Physics, University of Melbourne, Parkville, VIC 3010 Australia; 4 School of Engineering, RMIT University, Melbourne, VIC 3001 Australia; 5 Institute for Frontier Materials, Deakin University, Geelong, VIC 3216 Australia; 6 Australian Research Council Centre of Excellence for Integrative Brain Function, The University of Melbourne, Victoria, 3010 Australia; 7 Monash Biomedical Imaging, Monash University, Clayton, VIC Australia; 8 Department of Medicine (RMH), The University of Melbourne, Parville, VIC 3010 Australia; 9 Department of Psychiatry, The University of Melbourne, Parville, VIC 3010 Australia; 10 Florey Institute of Neuroscience and Mental Health, Parkville, VIC 3010 Australia

## Abstract

Today’s electronic devices are fabricated using highly toxic materials and processes which limits their applications in environmental sensing applications and mandates complex encapsulation methods in biological and medical applications. This paper proposes a fully resorbable high density bio-compatible and environmentally friendly solution processable memristive crossbar arrays using silk fibroin protein which demonstrated bipolar resistive switching ratio of 10^4^ and possesses programmable device lifetime characteristics before the device gracefully bio-degrades, minimizing impact to environment or to the implanted host. Lactate dehydrogenase assays revealed no cytotoxicity on direct exposure to the fabricated device and support their environmentally friendly and biocompatible claims. Moreover, the correlation between the oxidation state of the cations and their tendency in forming conductive filaments with respect to different active electrode materials has been investigated. The experimental results and the numerical model based on electro-thermal effect shows a tight correspondence in predicting the memristive switching process with various combinations of electrodes which provides insight into the morphological changes of conductive filaments in the silk fibroin films.

## Introduction

Bio-resorbable, environmentally friendly, transient integrated circuits represent a new class of electronics which pave the way towards new possibilities in the fields of environmental monitoring, biomedical diagnostics, sensors and the emerging field of electroceuticals. These new class of devices are capable of robust and reliable operation even when embedded within living tissue, and without causing deleterious inflammatory reactions^1^. Importantly, they can dissolve away after use, circumventing the need for their retrieval and disposal from the environment or in biological applications removal and reducing risk associated with added surgical procedures. Furthermore, these electronics can be designed with the desirable device lifetime transience via adaptation of the constituent materials, by which they can subsequently resorb through hydrolysis or metabolic action at varying timepoints^1^. Silk fibroin, extracted from the Bombyx Mori silkworm cocoon, represents an interesting novel biomaterial endowed with outstanding mechanical, electrical and optical properties^2–5^. Silk fibroin has been proposed to be an ideal material for producing a variety of high-performance biocompatible and flexible electronics devices, such as transistors, memory devices, and optical and optoelectronic components^3–6^. In addition, its high solubility in water, with adjustable dissolution rates^7,8^ via the use of various encapsulating materials such as polycaprolactone^9^ or thermally embossed and laminated silk fibroin^2^ represent another distinct advantage for environmentally friendly disposable electronics.

To date, there have been many attractive reports of biocompatible and bio-resorbable transient integrated electronics where silk fibroin has been used as passive component. It has been employed as substrate or conformal platform in the building of functional solid-state devices such as transistors, RFIDs and micro electrode arrays in field of bioresorbable, implantable applications^3,4^. It is also worth mentioning that there are other biomaterials such as egg albumen^10^, PMMA/PHEMA^11^, sericin^12^, and eumelanin^13^ that have been used to construct memristive devices. Interestingly, silk has been shown to exhibit resistive switching characteristics as well and has been used as an active building block of various biocompatible and flexible memristors fabricated on solid substrates such as glass, polythioesters and silicon^6,14^. However, the full resorbability of such silk based memory resistors, which plays an important role in their full biodegradability, remains a challenge with respect to fulfilling the demands of environmental friendly and sustainable electronics. In this work, we demonstrate for the first time a fully bioresorbable high density memristor configured as crossbar arrays which is fabricated on solution processable substrate, Poly-(vinyl alcohol) (PVA). This class of memristor provides a completely water soluble and fully resorbable component for emerging implantable and/or disposable electronics. The PVA substrate is used due to its superior elasticity (Young modulus of 13.5 GPa)^15^, tuneable solubility in aqueous solution^16^ and ease of preparation. This substrate can be prepared without additional post-treatment whereas crystallised silk fibroin based substrate not only requires an extended period to achieve the desirable mechanical strength for the substrate, it limits the transient capabilities of the fabricated device due to the insolubility of crystallised silk fibroin^17,18^.

There has also been limited attention in understanding the switching mechanism of silk based memristors which could offer more comprehensive information about optimum electrode combinations or the physiochemical alteration of the silk fibroin. In recent studies, different bipolar switching mechanisms have been proposed for the silk fibroin based memristive devices. M.K. Hota *et al*. describe a mechanism associated with a localised oxidation and reduction of silk fibroin protein chains, leading to an increase or decrease in resistance^6^. In contrast, Wang *et al*. explain the switching mechanism as the typical electrochemical metallisation memory (ECM) in which metal ions from the oxidation of the active electrode migrate through a solid electrolyte and electro-crystallises to form conductive filaments during the SET cycle^18^. The RESET cycle involves localised heating on these conductive filaments via joule heating leading to its dissolution. However, we believe there has yet to be substantial work in exploring the effects of various electrode materials on the silk fibroin memristor and how it relates to the electrical performance. Up to now, several studies have been carried out on silk based memristors which are fabricated based on various material combinations with the underlying switching mechanism having been interpreted inconsistently as is indicated in Table S1.

In this paper, we report on the development process of the bio-transient memristive device fabricated on a solution processable PVA substrate with a focus on the effects of electrode materials on the switching mechanism. For comparison and simplicity purposes, we have also fabricated similar electronics on glass substrates. The findings on the impact of different combinations of active and inert electrodes with respect to the electrical characteristics and performance of the crossbar memristive device strongly support the electrochemical metallisation model. To further elaborate on our experimental observations, we investigated the influence of silk fibroin crystallinity in addition to measuring the electrochemical response over a variety of active electrodes. Moreover, a mathematical model of the growth and dissolution of conductive filament is proposed and simulated to elucidate the physical mechanism behind the observed phenomena. Our results provide important, novel insights into the development of future robust and non-volatile bio-transient memory devices.

## Results

**Figure 1:**
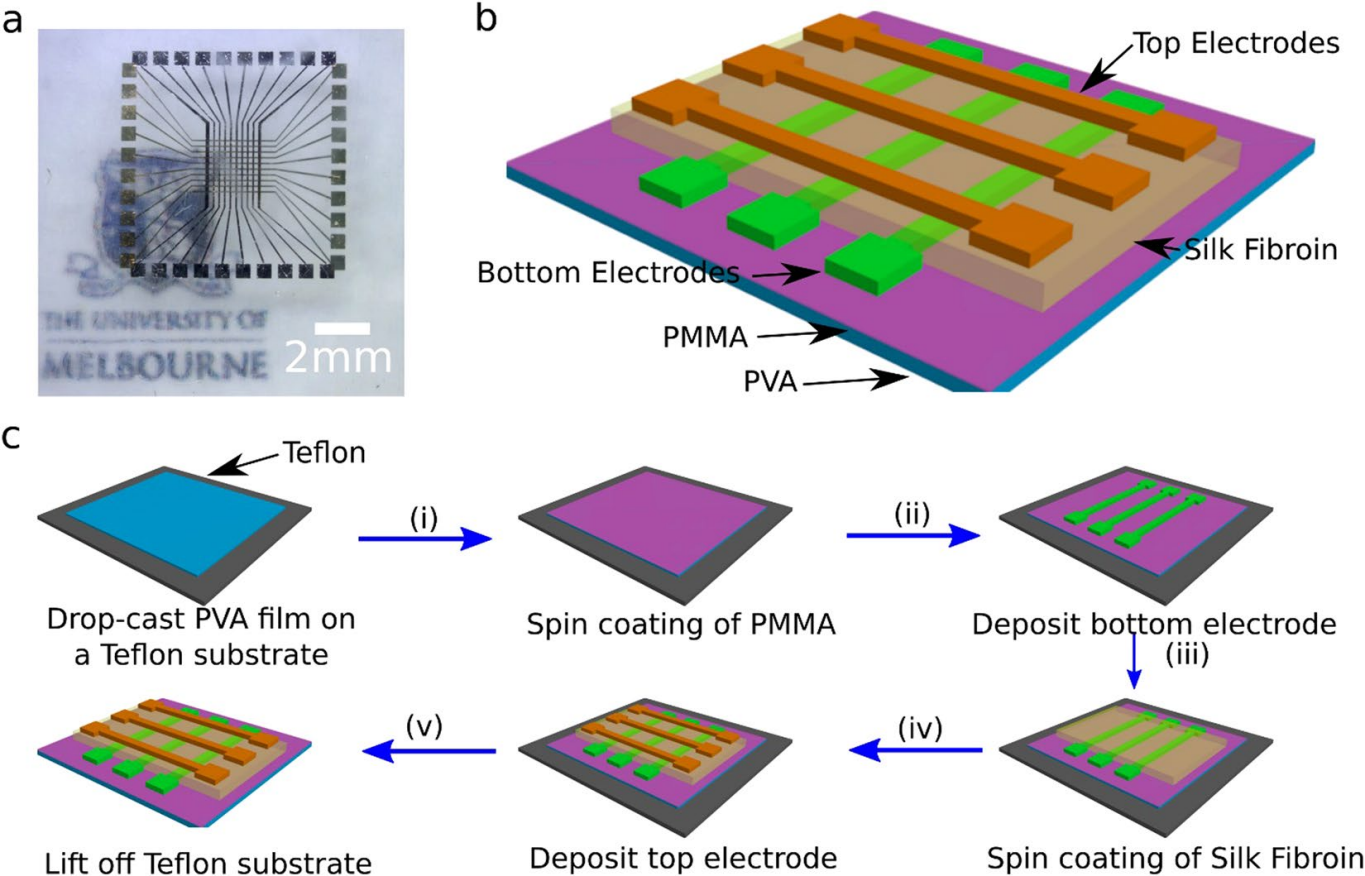
Device structure and fabrication process of the silk fibroin memristor. (**a**) Photograph of an Au-Silk Fibroin-Pt crossbar memristor device fabricated on a PVA film. (**b**) Schematic illustration of the fabricated crossbar memristive device. (**c**) Flowchart illustrating the fabrication process for a bio-resorbable and biodegradation crossbar memristive device. The PVA substrate is drop-casted on a Teflon surface to ease substrate lift off and followed by the spin coating of PMMA to prevent dissolution during silk fibroin deposition. The bottom gold electrodes and top platinum electrodes are patterned via shadow mask e-beam evaporation whilst the switching layer is a solution processed silk fibroin. Cross sectional interfacial structure of the device is shown in Fig. S1.

We have developed a crossbar memristive device that consists of silk fibroin switching layer stacked between an active electrode and an inert electrode supported on a spin-cast substrate of PVA. Figure 1a shows a crossbar memristor fabricated with 50 nm gold (Au) and 50 nm platinum (Pt) electrodes to ensure bio-transient capabilities. Practically, a thin Cr layer (~ 10 nm) is introduced in the interface between the PVA substrate and the electrodes to promote adhesion which has demonstrated no increase in cytotoxicity. It has been shown that Cr in its pure metallic form does not contribute to any adverse health effects^19–22^. The device consists of a ten by ten active and inert electrode arrays. A shadow mask along with e-beam evaporation was used to pattern and deposit the active electrodes arrays on the various substrates primarily glass or PVA and followed by spin coating silk fibroin films. Figure 1b and c display the temporal device fabrication sequence.

**Figure 2:**
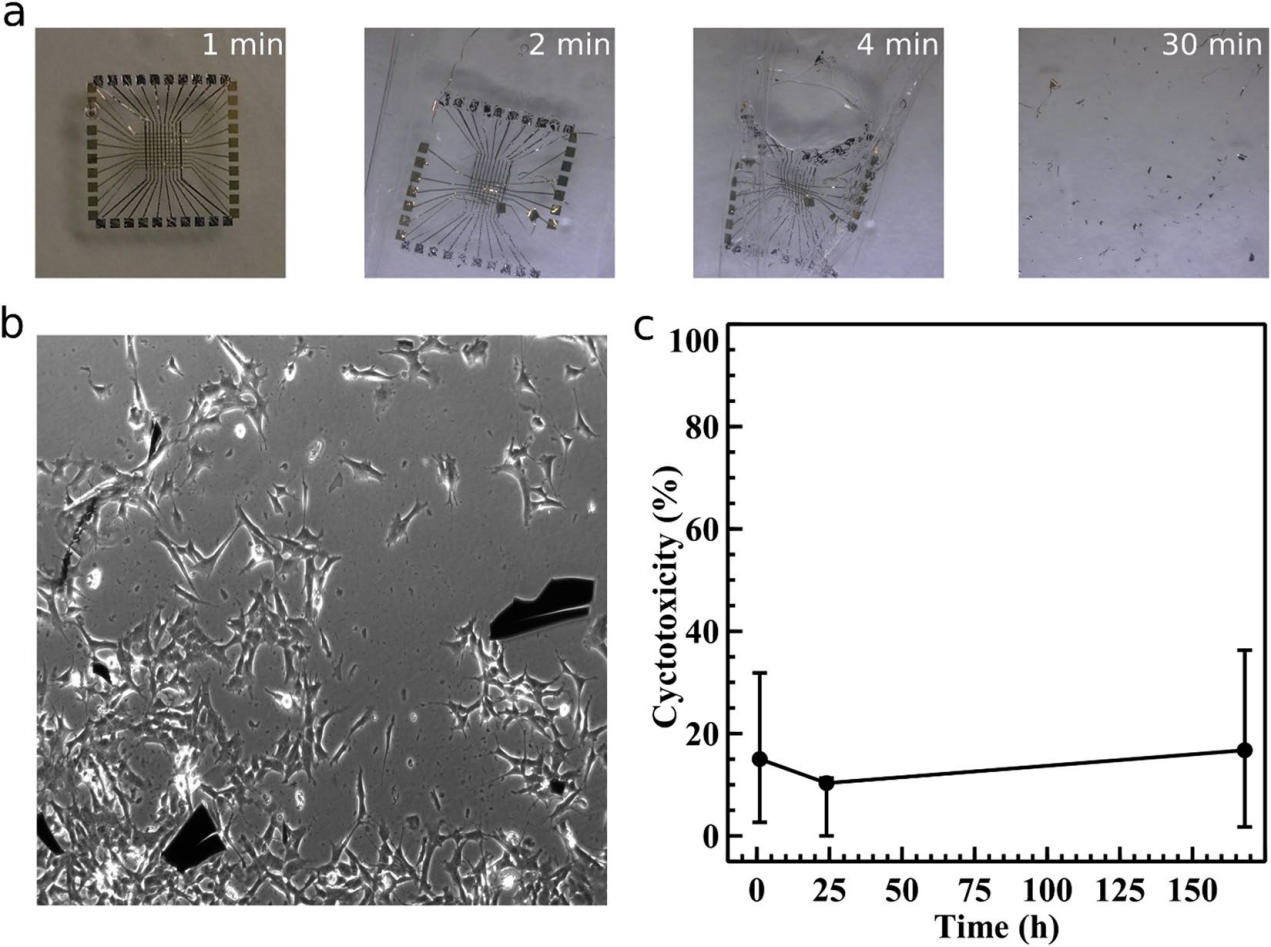
Device bio-resorbability and cytotoxicity characteristics. (**a**) Time sequence of the dissolution of the crossbar memristive device in DI water under ambient conditions. (**b**) Image of SY5Y neuroblastoma cells proliferating in the presence of the bio-resorbing memristors for 168 hrs. 10^3^ cells/well was selected to be the standard seed cell number in the cytotoxicity test to study the effects of the constituent materials. (**c**) Time dependent curve for cell viability assessment in SH-SY5Y neuroblastoma cells in direct exposure to the memristive device. Indicated values are means of 8 experimental sets.

The transient characteristics were evaluated by the immersion of the device in de-ionised (DI) water under ambient conditions. Figure 2a shows a time sequence of images illustrating the dissolution process of the crossbar patterned memory. The crossbar device showed rapid disintegration within 2 minutes and complete dissolution within 30 minutes. This dissolution process is due to silk degradation in water. This characteristic is crucial for *in vivo* applications as the silk fragments subsequently degraded through proteolytic mechanisms and resorbed without leaving traces of the fabricated device^23^. It is important to note that silk fibroin, used as the functional constituent, is a biocompatible product, approved by the Food and Drug Administration (FDA) for a number of different clinical applications^24^. For this investigation, we chose to assess cytotoxicity, a component of biocompatibility, of all the materials and fabrication steps of our fabricated device via the lactate dehydrogenase cytotoxicity (LDH) (Promega, US). We have employed the lactate dehydrogenase cytotoxicity (LDH) (Promega, US) assay to evaluate any cellular membrane damage associated with the memristor. This enabled us to evaluate any gross cellular damage, related to cytotoxicity as determined by the release of LDH from the cytoplasm into the surrounding media following cell death. LDH levels were then detected via colorimetric assay through oxidation of a coloured substrate and read in a plate reader at 490 nm^25^. We also included positive controls where all cells were lysed giving a maximal response as well as negative controls containing non-exposed cells and background absorbance measured from the culture media. Figure 2b shows the cultured cells in a dish in which a typical memristive device was fragmented and dissolved. Figure 2c graphs the levels of apparent cytotoxicity when the fabricated memristor was introduced into SH-SY5Y cells cultures as measured using LDH assay. The results indicate that the presence of the fragmented device in the culture medium did not cause any obvious cytotoxicity.

**Figure 3:**
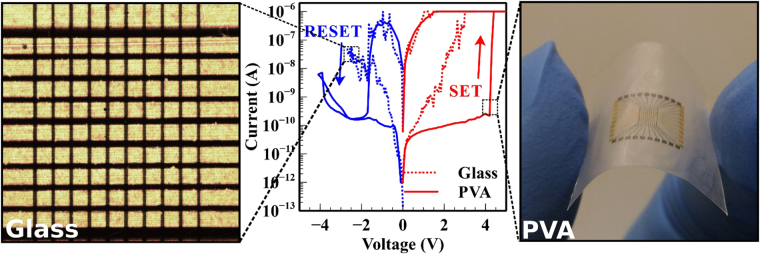
Electrical characteristics of the silk fibroin memristive device. Measured IV characteristics of the crossbar memristive device fabricated on a glass substrate and a PVA substrate (centre). The Pt electrodes are set at 0 V and the Au electrodes are subjected to a ± 5 V, 1 Hz triangle voltage waveform with a current compliance of 1 µA at room temperature. Photograph of a Au-Silk Fibroin-Pt crossbar memristive device on a glass substrate (left). Photograph of a free-standing Au-Silk Fibroin-Pt crossbar memristive device on a PVA substrate (right).

Next, we investigated the electrochemical properties of the silk fibroin memristors by performing systematic studies. The working principle of the proposed memristive device involves a redox reaction on the electrodes as well as ionic migration within the insulator. On application of the SET voltage, oxidation occurs on the active electrode. The subsequent metallic ions migrate towards inert electrode induced by the applied electric field. This leads to electro-crystallisation and the formation of conductive filaments which result in the rapid reduction in bulk resistance. This transition is termed the SET process. On the other hand, joule heating is raised due to enhanced thermal conductivity of the silk and large current density in percolated filamentary path which causes the dissolution or rupturing of these filaments while applying a RESET voltage, resulting in the rapid increase in bulk resistance and is termed the RESET processs^26,27^. In a typical bipolar memristor device, the SET and RESET voltage are of the opposing polarity^28^. To examine the current-voltage (IV) relationship, a triangle voltage waveform of amplitude 5 V was swept at a frequency of 1 Hz. Figure 3 shows the IV characteristics of the Au-Silk Fibroin-Pt memristive device on two different substrates (namely glass and PVA shown in Fig. 3) in which the Au and Pt electrodes serve as the anode and cathode respectively. The fabricated memristive device exhibited non-volatile bipolar switching behaviour where a high resistance state (HRS) and low resistance state (LRS) can be observed via the application of voltage which was also shown to be reversible within the life-cycle of the device. The voltage was swept in the sequence of 0 V → 5 V → 0 V → −5 V → 0 V with a current compliance level set at 1 µA. By subjecting the active Au electrode to an increasing voltage, the memristor (fabricated on a glass substrate) exhibited a notable change in resistance to a LRS of 5 × 10^4^ Ω which was recorded during the SET cycle at 3 V. Reversing the polarity of the applied voltage, a RESET cycle was realised in which an abrupt change to a HRS of 0.2 × 10^9^ Ω occurred at −1.7 V. Remarkably, the fabricated device showed a HRS/LRS ratio of 10^4^ ~ 10^6^ which provides a significantly large margin for differentiating the on to off states (“1” or “0”). The memristor fabricated on a PVA substrate showed a transition from the HRS of 2 × 10^10^ Ω to the LRS of 3 × 10^6^ Ω and vice versa, with a HRS/LRS switching ratio of 10^2^ ~ 10^4^. The SET cycle for the PVA substrate memristor device occurred at a higher voltage of 4.4 V whereas the RESET cycle also occurred at −1.7 V. The differences observed in memristive switching between the PVA and glass substrates can be attributed to the non-uniformity of the silk fibroin films as shown by AFM measurements in Fig. S2. These non-uniformities in silk fibroin thickness resulted in the observed dissimilar resistive switching characteristics. The rough surface of film PVA originated from the Teflon support substrate which impedes the uniform viscous flow of the silk fibroin film leading to a larger thickness and hence, higher operating voltage of the memristor.

**Figure 4:**
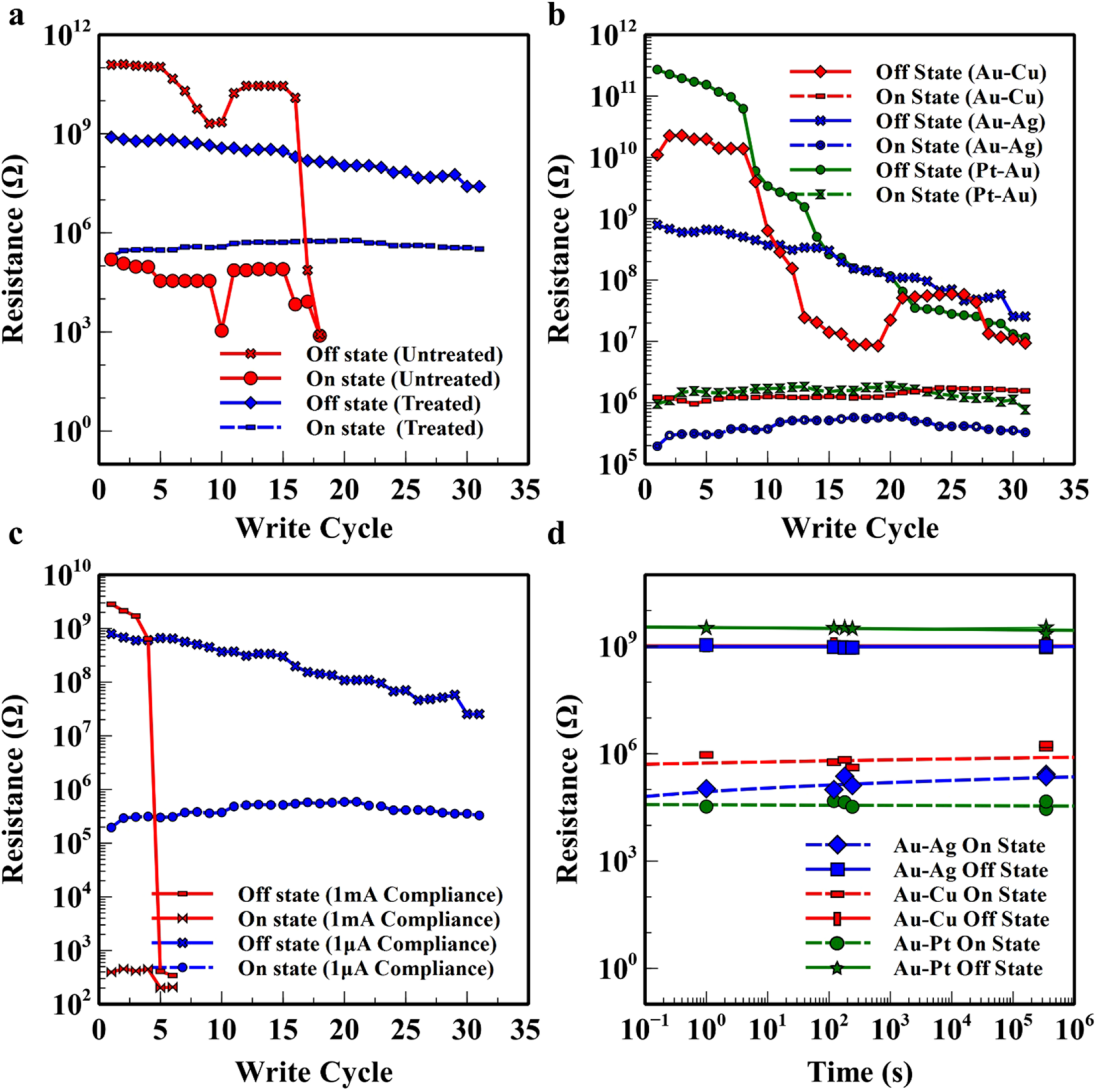
Endurance characteristics and performance of the silk fibroin memristive device. (**a**) Endurance characteristics for the untreated and the water annealed Au/SF/Ag memristive devices. (**b**) Endurance performance of the fabricated memristive device with various combinations of electrode materials, namely, Au-Ag, Au-Pt and Au-Cu. (**c**) Endurance characteristics for the Au/SF/Ag device with current compliance level of 1 mA and 1 µA. (**d**) State retention time for the fabricated memristive device with various Au-Ag, Au-Pt and Au-Cu electrode combinations over the course of 96 hrs.

One of the key criteria for assessing the memristor performance is the stability of the two resistance states. It has been found that the water annealing process induces crystallinity in the silk fibroin material when incubated in a high humidity environment. The introduction of water molecules disrupts intermolecular cohesive forces between the protein chains and reduces steric hindrance which increases mobility of non-crystalline domains in protein^29^. Therefore, the memristors were treated through a water vapour annealing process to tailor their water solubility and stability; an important step in controlling how fast the devices would dissolve. The water treated and untreated devices were subjected to their respective SET/RESET conditions sequentially and the states were read at a voltage of 0.4 V. Devices that had undergone the water annealing treatment exhibited superior endurance characteristics with respect to the untreated devices as depicted in Fig. 4a. Fourier transformed infrared (FTIR) spectroscopy was performed on both pre- and post-water annealed silk films to verify the degree of crystallinity. Fig. S3 illustrates the infra-red adsorption spectra of the fibroin film. The absorbance strength ratio of the spectrum 1265 cm^−1^ and 1235 cm^−1^ (A_1265_/A_1235_) is commonly used to determine the crystallinity index^30–33^. The results show that the water annealed silk fibroin films obtained a higher crystallinity index of 0.80 whereas the untreated films had an index of 0.71. When forgoing the water annealing process, the fabricated devices had a higher tendency to form pinholes in the silk, creating short circuits, during the metal deposition process. This increase in pinholes was attributed to the poorer mechanical strength of the untreated silk fibroin film^34^ and the formation of cracks in the film as is shown in Fig. S4. Thus, the water annealing treatment improves the device yield (the number of fabricated devices with no formation of pin holes) from 41.6% to 81.9% when tested on the 200 fabricated devices.

The performance of the bio-resorbable memristive device with different combinations of active and inert electrodes, namely Au-Pt, Ag-Au and Cu-Au was also investigated. The fatigue resistance test and retention time test were performed to verify the stability and durability. The results, shown in Fig. 4b, suggest that Au-Pt and the Cu-Au combinations experience larger degradation in off resistance retention after the 20^th^ cycle in comparison to the Ag-Au combination. Interestingly, the proposed switching mechanism involving the oxidation and reduction of the localised protein chain as discussed in the literature^6^ and would be dependent on the work function of the electrodes used. The work function of copper, gold and silver were 4.7 eV, 5.1 eV and 4.73 eV respectively^35^. We have observed large variations in the memristor performance for the Ag-Au and Cu-Au electrode combinations although the copper and silver electrodes have similar work function. This implies that the redox reactions of cations at the interface of silk/electrode plays a critical role in the switching mechanism in addition to the ionic mobility of cations. Overall, the endurance characteristics of the memristive device using various combinations of active and inert electrodes indicate excellent reversibility/reproducibility and acceptable stability prior to the 20^th^ read/write cycle. Current compliance plays an important role in overall device lifetime in addition to the on and off state resistance. We measured that the memory margin increased significantly to an on/off ratio of 10^8^ by increasing the current compliance toward a higher level. Fig. 4c illustrates the measured on/off state resistance for an Au-Ag memristor with two current compliance levels of 1 µA and 1 mA. It was observed that the higher current compliance (1 mA) results in a reduced lifespan of two write cycles whilst the lower compliance current (1 µA) permitted for increased lifespan exceeding 30 write cycles. This behaviour is attributed to joule heating effect which deteriorates the silk fibroin switching layer through intense localised heating^26,27^. It is worth noting that, as shown in Fig. S5, the initial device switching characteristics differ from subsequent switching cycles. This is due to initial electroforming process which is commonly observed in ECM type memristive devices^36^. It is believed that morphological change in the electrolyte occurs upon first metallic filament formation leads to a pre-configured electroforming path as an easy transport channel for all upcoming switching processes. Subsequently, the retention performance was evaluated where the fabricated devices memory state was SET and then subjected to the read voltage over the course of 96 hrs (Fig. 4d). The stability of the memory retention demonstrates the potential of the proposed device in non-volatile memory applications.

**Figure 5:**
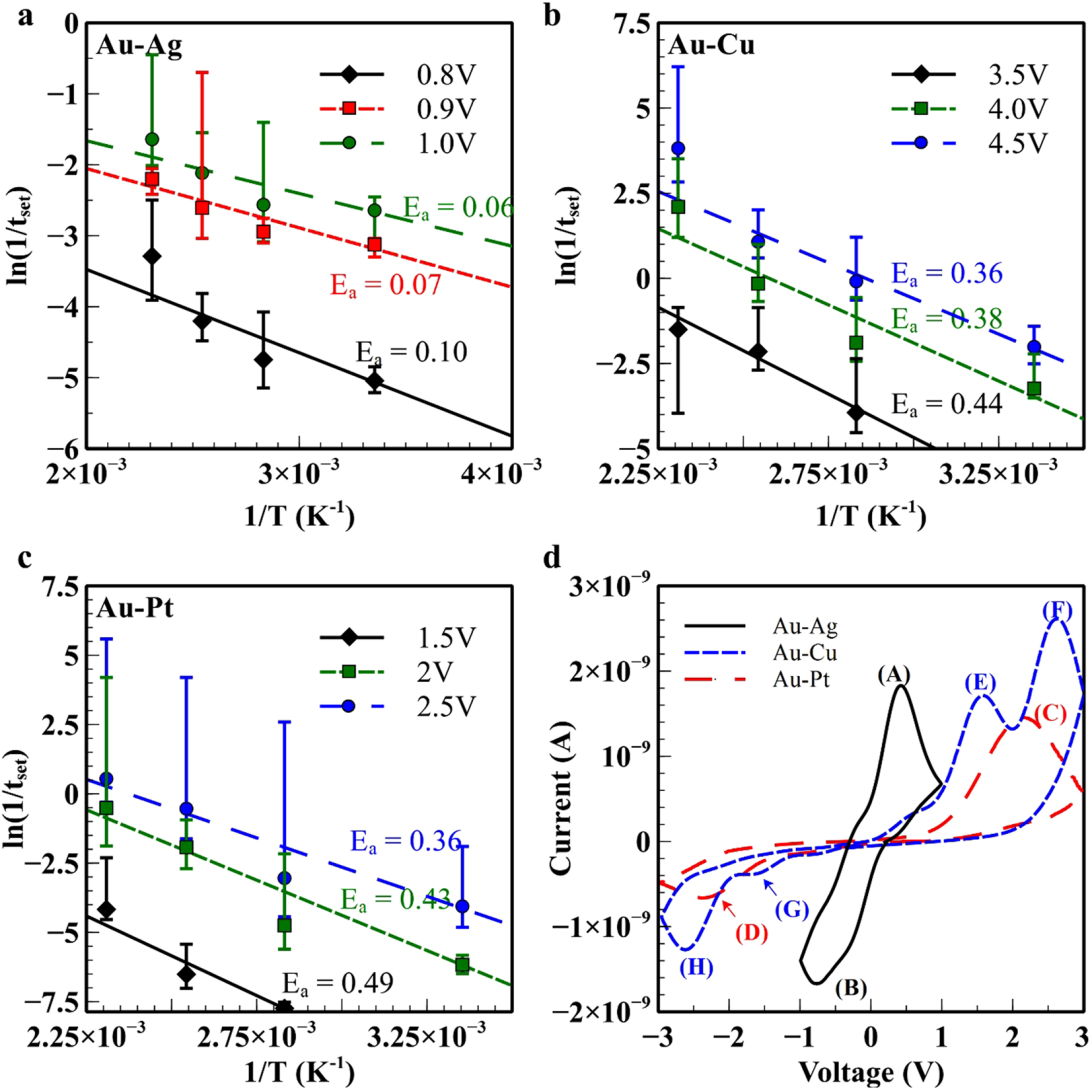
State transition time-temperature characteristics of the memristive device and corresponding Arrhenius plot. Arrhenius plot of the measured transition time at different electric potential with various combination of active-inert electrode material: (**a**) Au-Ag with an applied voltage of 0.8 V, 0.9 V and 1.0 V (**b**) Au-Cu with an applied voltage of 3.5 V, 4.0 V and 4.5 V (**c**) Au-Pt. with an applied voltage of 1.5 V, 2 V and 2.5 V. These measurements were extracted from the chrono-amperometry measurements with varied ambient temperature of 25 °C, 80 °C, 120 °C and 160 °C. Indicated values are mean of 10 devices each. See supplementary materials for details. (**d**) Comparisons of the cyclic voltammograms for various electrodes as shown in the legends. The scan speed is 50 mV/s.

The physicochemical properties of the switching characteristics for silk fibroin memristor play an important role in the functional characteristics of the device. Chrono-amperometry and cyclic voltammetry (CV) techniques were employed to study the effects of the various electrodes on the overall performance and electrical characteristics of the memristive device. Fig. S6, Fig. S7 and Fig. S8 illustrate the chrono-amperometry results in which the SET transition time was determined to estimate the Arrhenius parameters. Figure 5a–c shows the Arrhenius plot for varying ambient temperatures ranging from 298 K to 433 K with various electric potentials applied to the memristive device. Based on the chronoamperometry measurements, it was observed that the state transition time decreases exponentially with both increasing ambient temperature and with increasing applied voltage. The extraction of the effective activation energy, E_a_, was calculated via the $\mathrm{ln}(1/{t}_{set})={e}^{-{E}_{a}/{k}_{B}T}$ expression, where k_B_ is the Boltzmann constant, T is the ambient temperature and t_set_ is the SET transition time and used to calculate the zero-potential activation energy and the electric potential induced barrier lowering parameter. Figure 5d shows the CV measurements for the silk fibroin memristive device for various electrode materials with an area of 41μm × 41μm as depicted in Fig. S9. These measurements were performed with sweep rate of 50 mV/s whilst the potential window was restricted to a lower amplitude range to prevent the onset of the resistive switching process. The observed peak currents in the cyclic-voltammetric curves are attributed to the dissolution of active electrode material in silk via the oxidation to cations followed by deposition of reduced (neutralized) cations at the silk-counter electrode interface^37^. It should be mentioned that the geometrical characteristics of the proposed memristor device requires the omission of the reference electrode which is typically used in CV measurement to ease the identification of the electrochemical reactions. However, by considering the thermodynamic stability of the ionic species, it is possible to deduce the corresponding partial electrochemical reaction^37^. Referring to the CV measurement for the Ag-Au electrodes, the peak (A) and (B) can be associated with the direct oxidation of Ag to Ag^+^ and reduction of Ag^+^ to Ag respectively. Similar assignments of recorded peak currents to their respective reactions at the electrode interface have been previously reported^37–39^. In this configuration (Ag/Au), the oxidation of the Au electrodes has not occurred because of the consistent ability of our device to retain the off state when subjected to the negative polarity (as shown in Fig. S5). For the memristor device with Au-SF-Pt configuration, peaks (C) and (D) are proposed to be associated with the direct oxidation of Au to Au^+^ and reduction of Au^+^ to Au respectively due to the similarly occurrence of peak current with respect to the Ag electrodes. In the case of the Au-SF-Cu memristor, peaks (E) and (F) can be linked to the oxidation of Cu to Cu^+^ and Cu to Cu^2+^ respectively. The peak (G) is associated with the reduction reaction Cu^+^ to Cu and peak (H) is linked to the reduction reaction of either Cu^2+^ to Cu^37,40^. Other partial reactions of the ionic species such the reduction of Cu^+^ to Cu and Cu^2+^ to Cu^+^ could also have occurred^40,41^. The current peaks of these partial reactions may be obscured by the lower resolution of the two-electrode cyclic voltammetry method. In addition, these current peaks have been observed to fluctuate for different voltage sweep cycles as concentration of these ionic species constantly changes^40^. The studies with different electrode combinations suggest that the switching characteristics are highly influenced by the type of electrodes used which would deviate from the findings that the reduction or oxidation of localised silk fibroin protein chains are the primary factors for the resistive alterations^6^. Furthermore, our studies demonstrate the feasibility of tuning the operating voltage of the memristor device by the selections of electrode material.

Based on the experimental data, we have observed that the Ag electrodes exhibited the highest likelihood for the formation of conductive filament followed by the Au and Cu electrodes. These observations differ from the expected outcome as would be predicted by their standard electrode potential in which the Cu electrode should have the highest tendency for ionic migration. The likely reason for this phenomenon is that the electrolytic migration rate of metal ions in a thin insulator is limited and can be determined by the following expression:^42,43^

1 $${\rm{t}}=\frac{{\rm{Nz}}\,{{\rm{x}}}^{2}{\rm{r}}}{2\,{{\rm{n}}}_{{\rm{c}}}{\rm{V}}}$$

where t is time required for the ions to traverse through a thickness of x, N is the cations density, z is the charge of the cations, ρ is the resistivity of the insulator, n_c_ is the transport number of the cations and V is the applied voltage. From Equation 1, metal elements with higher charge cations will require a longer time to migrate along the insulator, resulting in a longer state transition time. Similarly, the transition time can also be reduced by increasing the applied voltage between these electrodes. Thus, the Cu electrode will typically exhibit higher state transition voltage compared to the Ag and Au electrodes to achieve similar state transition time. This is presumably due to the higher thermodynamically stable oxidation state of +2 for the copper element which has been observed in various thin insulators, such as vitreous silica and quartz^42^. Thus, for circumstances in which the electrolytic process is limited by the charge transfer kinetics, e.g. an insulator with low ionic conductivity, the stable oxidation state of the metal ions of the electrodes seems to be the key factor determining the electrical properties and resistive state stability of the memristor device whereas the standard electrode potential of the metal electrodes has less pronounced effect.

Based on our findings, the electrochemical metallisation model proposed for a silk fibroin memristor device can be described in several steps, beginning with the electrochemical dissolution of the active electrodes into their respective ionic species, followed by ionic migration of the cations towards the inert electrodes under high electric field and lastly, the electro-crystallisation of cations to form conductive filaments. On the other hand, the dissolution of these conductive filaments involves the thermally assisted dissolution of metals via joule heating under high electric field of the opposing electric potential. The opposing electric potential ensures the cessation of filamentary growth and promotes the rupturing of these conductive filaments. In addition to silk fibroin, it has been demonstrated that typical dielectrics or insulators such as silicon dioxide, titanium oxide and nickel oxide exhibits electrolytic characteristics provided they are sufficiently thin, usually ranging from tenths to hundredths of nanometres^38^. The SET process involves the application of sufficiently high positive electric potential on the active electrode which leads to an oxidation process on the active electrode-insulator interface as describe by reaction 2:^44^

2 $${\rm{M}}\leftrightarrow {{\rm{M}}}^{z+}{+\text{ze}}^{-}$$

where M, M^z+^ and z denotes the metal atoms, metal ions and the charge of the ionic species respectively. It is suggested that primarily Ag^+^ and Au^+^ ions are involved for Ag-Au and the Au-Pt electrode combination respectively whereas the Cu^+^ and Cu^2+^ ions are involved for the Cu-Au electrode combination. The cations formed from this reaction proceeds to migrate towards the inert electrode due to the high electric field. Lastly, these cations are gradually reduced and electro-crystallised on the inert electrodes and forms conductive filaments which are associated with the SET process. Cyclic voltammetric studies in Fig. 5d have revealed the relevant ionic species corresponding to each of the oxidation and reduction process for various electrode combinations.

On the other hand, the RESET process involves the application of a negative electric potential in which these conductive filaments undergo thermal dissolution as well as the reduction to their ionic form, indicated by reaction 3, and as suggested by the cyclic voltammetric studies.

3 $${{\rm{M}}}^{{\rm{z}}+}+{{\rm{ze}}}^{-}\leftrightarrow {\rm{M}}$$

An interesting observation is that metallic ions with higher oxidation states may require the application of a higher electric potential to initiate the oxidation process and can be described by Equation 1. To facilitate the oxidation process of the active electrode, a complementary reduction process has to occur on the inert electrode–insulator interface^38^. In the case of a thin film silk fibroin layer, we believe that water content within the silk fibroin film, similar to that of silicon dioxide^37,40,41^ and tantalum oxide^45^, is involved in the reduction process as shown below:

4 $$2{{\rm{H}}}_{2}{\rm{O}}{+\text{2e}}^{-}\leftrightarrow 2{\rm{O}}{{\rm{H}}}^{-}+{{\rm{H}}}_{2}$$

5 $${{\rm{2H}}}_{2}O+{O}_{2}+{{\rm{4e}}}^{-}\leftrightarrow {\rm{O}}{{\rm{H}}}^{-}$$

Typically, silk fibroin film has an inherent water content of 7.5% under ambient conditions^7^. Several proposed candidates as depicted by reaction 4 and reaction 5 are used to account for these reduction processes with which may not be the full representation due to the possibility of various other intermediary reactions^44^. These reactions are highly dependent on the chemical properties of the insulator film as well as the adsorption properties of the electrodes used^38^. Numerical modelling of these phenomena can provide an insight of the physical changes within the silk fibroin layer. The ionic migration velocity in an insulator under applied electric field can be expressed as:^43^

6 $${\rm{v}}=\frac{{{\rm{n}}}_{{\rm{c}}}{\rm{V}}}{{\rm{N}}\,{\rm{z}}\,{\rm{\rho }}\,{\rm{x}}}$$

where x is insulator thickness, N is the cation density, z is the charge of the cations, ρ is the resistivity of the insulator, n_c_ is the transport number of the cations and V is the applied voltage. The kinetic mechanism of the electro-crystallisation process can be described in form of:^46^

7 $${\rm{J}}({\rm{t}})={Z}_{0}{\rm{W}}{{\lambda }}^{-1}{{\rm{e}}}^{-{\rm{G}}{\rm{\Delta }}({{\rm{n}}}_{{\rm{c}}})/{\rm{R}}{\rm{T}}}$$

where J(t) is the rate of nucleus formation, Z_0_ is the density of nucleation sites, W is the frequency of nucleus attachment, λ^−1^ is the Zeldovich factor and G^Δ^(n_c_) is the energy barrier for the conversion of n_c_ number of ions into their solid phase. Despite the theoretical accuracy, it is difficult to correctly ascertain the parameters used in equations 6 and 7. Conversely, the Arrhenius equation provides a simple closed form expression with similar form for modelling these behaviours and will be fitted to the experimental data.

Here, we introduce a numerical model to further corroborate on the proposed switching behaviour for silk fibroin based memristive and prove mode insight into understanding of the switching mechanism based on the morphological changes of the conductive filament. To facilitate the finite element simulation of the dissolution and growth of the conductive filament, a cylindrical conductive filament is proposed to describe the initial nucleation of the conductive filament which will be used as basis of the resistive transition. The simulation model assumes that the initial electroforming process has occurred and consists of a silk fibroin layer which encapsulates a cylindrical filament of diameter 2 nm in agreement with surface resistivity observation using a conductive atomic force microscopy^6^. The top and bottom electrodes were expressed as single dimensional lines and as the active electrodes, assumed to be an infinite source of cations. This simulated geometry is as shown in Fig. S10a. A simulation model based on the numerical solution for joule heating (Equations 8–11) as well as the growth or dissolution of the conduction filament using Equations 12–13 are performed with simulation package (COMSOL Multiphysics, MATLAB) to realize the state transition behaviour of the memristive device. The simulation process flow is depicted in Fig. S10b. The following expressions are used to explore the joule heating characteristics for the conductive filament:

8 $${\rm{k}}\text{'}{\nabla }^{2}{\rm{T}}=-\gamma \overrightarrow{{\rm{J}}}\cdot \overrightarrow{{\rm{J}}}$$

9 $$\gamma ^{\prime} =n\gamma $$

10 $${\rm{k}}\text{'}={\rm{n}}\,{\rm{k}}$$

11 $${\rm{J}}=\frac{1}{{\gamma }\text{'}}\nabla {\phi }$$

where k is the thermal conductivity, γ is the resistivity, T the temperature of the conductive filament, φ the temperature gradient and J is the current density. An additional fitting parameter n is defined to account for the effects due to the development of multiple conductive filaments as well as any non-uniformity, defects and voids in the filaments itself. Based on the Wiedemann-Franz law^47^, the thermal conductivity is assumed to exhibit a similar proportionality to n-parameter. These differential equations only accounts for the steady state heat transfer mechanism since the migration of ions, the electro-crystallisation process and the filamentary dissolution process are relatively slower than a typical thermal system^48^. An Arrhenius fitting equation is employed to describe the growth and dissolution of the conductive filaments as they provide a simple yet close approximation of the mathematical behaviour of the Equation 6 and 7. The electric field-assisted migration of ions in the silk fibroin layer exhibited a linear dependency with respect to the effective activation energy, E_a_^49,50^, as shown in Fig. 5. Based on these Arrhenius plots, the extracted zero-potential activation energy, E_0_, were 0.25, 0.69 and 0.71 whereas the energy barrier lowering parameters, α, are 0.19, 0.13 and 0.08 for the electrode combinations Au-Ag, Au-Pt and Au-Cu respectively.

12 $$\frac{{{\rm{dr}}}_{{\rm{set}}}}{{\rm{dt}}}={{\rm{A}}}_{{\rm{set}}}{{\rm{e}}}^{-\frac{{{\rm{E}}}_{0}-\alpha {\rm{V}}}{{\rm{RT}}}}$$

13 $$\frac{{{\rm{dr}}}_{{\rm{reset}}}}{{\rm{dt}}}={{\rm{A}}}_{{\rm{reset}}}{{\rm{e}}}^{-\frac{{{\rm{E}}}_{0}}{{\rm{RT}}}}$$

where r is the growth or dissolution radius of the conductive filament, A is pre-exponent fitting parameter, E_0_ is the zero-potential activation energy, R is the Boltzmann constant, T is the temperature, the α is the barrier lowering parameter and V is the applied electric potential. The material parameters for the silk fibroin film, described in Table 1, were used in the simulation. The numerical procedure includes two main steps where the coupled Equations 8–11 were solved based on a 2D symmetrical model followed by the extraction of the temperature and electric potential profile along the surface of the conductive filament. These data determine the growth or dissolution rate, governed by Equations 12–13, and are used to update the geometry of the filament as is illustrated in the flowchart in Fig. S10B. The numerical methods correctly replicated the experimental results when the fitting parameters described in Table S1 were used.

**Table 1:** Electrical, thermal and mechanical properties for Silk Fibroin. The following material properties are used in the simulation model to replicate the experimental data.

Material Properties	Value	Ref
Thermal conductivity (W/m K)	0.256	52
Density (kg/m^3^)	1398	53
Heat Capacity (J/g K)	0.134 + 3.696 × 10^−3^ T	54
Electrical Conductivity (S/m)	4.4 × 10^−13^	55
Relative Permittivity	6	56,57

**Figure 6:**
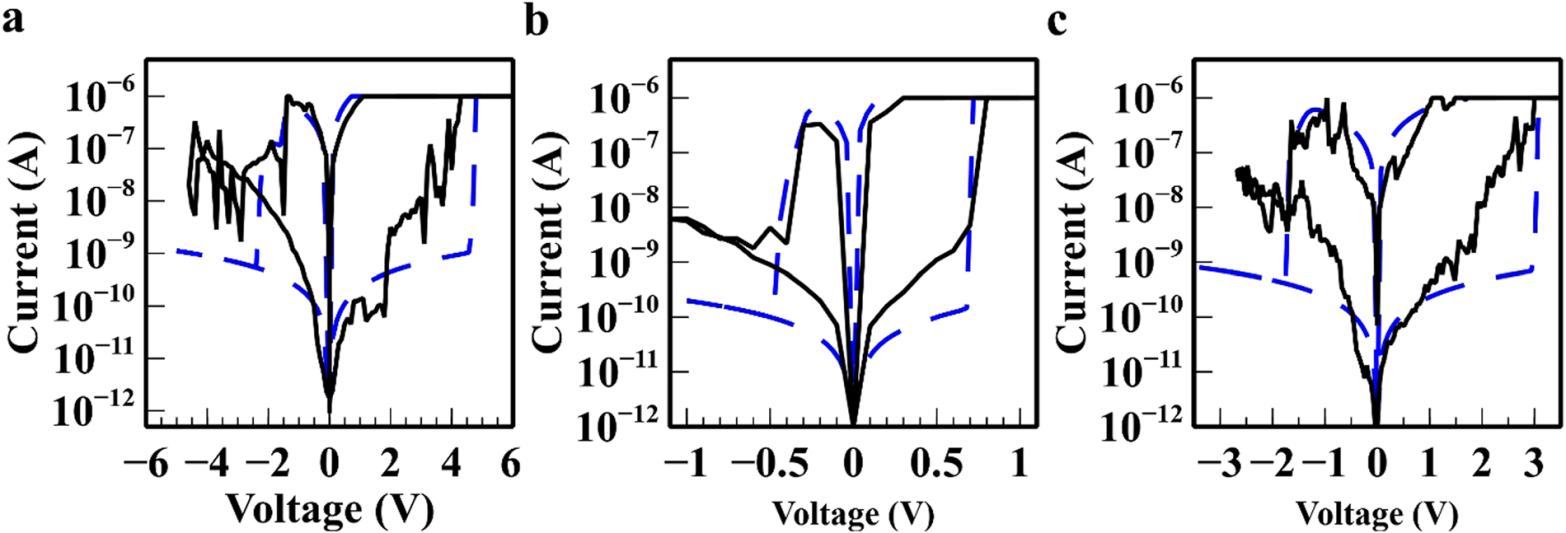
Comparison between numerical simulation and experimental IV characteristics of the set/reset process for different combinations of electrode material (**a**) Cu-Au electrodes (**b**) Au-Ag electrodes (**c**) Pt-Au electrodes. The blue dashed curve represents with experimental results; (the black solid line represents experiments while the dashed line shows simulation results). Geometrical and physical parameters are listed in Fig. S10 and Table S1.

Figure 6a–c shows the comparison between the simulated and experimental IV characteristics for the fabricated memristive device with various combination of electrode material. The numerical model predictions show good correspondence with the measured IV characteristics which substantiate their validity for these types of memristive device. The morphological changes in the conductive filament illustrate the device transition from a low resistance state to high resistance state and vice versa. Physical geometry of the conductive filament corresponding to their temperature distribution is illustrated in which Fig. S11A–D shows the reset transition with increasing negative electric potential whereas Fig. S11E,F shows the set transition with increasing positive electric potential. The proposed numerical model predicted a localised region of high temperature at approximately ~450 K. Several assumptions were made to simplify the simulation process which includes the cessation of filament growth following the formation of a continuous conduction bridge, non-active electrodes are ideally inert and formations of voids in the electro-forming or electrochemical process are ignored. The effects of dissolution due to joule heating during the set process are assumed to be negligible as observed from experimental data. It is observed that the rate of growth of these conductive filaments is in equilibrium with the rate of dissolution and that this translates to the stability of the on-state resistance. The bulk conductivity of silk fibroin has been increased to 400 μS/cm to account for the higher conductivity as observed from the experimental results following the electroforming process. This is presumably caused by the higher concentration of cations and residuals from ruptured conductive filaments in the silk fibroin film.

## Discussion

We have proposed and demonstrated an environmentally friendly and bio-resorbable memristive device fabricated by interlaying a silk fibroin layer, extracted from the Bombyx Mori silkworm cocoon, between an inert (Pt) and active (Au) metal electrode. The fabricated devices exhibit bipolar switching characteristics with an on/off ratio of > 10^4^, a lifecycle of > 30 and a state retention time of > 96 hrs. This device showed a physical transience of about 30 mins in DI water under ambient conditions. This is a critical step in the development of printable and bio-resorbable electronics which offers great potential to envisage fabrication of on-demand electronics that are used for tailored specific implantable devices and bio-sensors. Furthermore, various combinations of electrode materials were used to study their effects on the switching characteristics of the fabricated device as well as their respective physicochemical properties. Although the standard electrode potential of the metal electrodes does provide slight influences, it was observed that the oxidation states of the cations strongly affects the switching mechanisms of the fabricated memristive devices for systems with low ion mobility. In general, it can be concluded that cations with high stable oxidation states require higher SET/RESET voltage. Thus, the selection of electrode combinations to form the memristive device must account for these factors to avoid haphazard switching characteristics which may lead to erroneous retention of states. A numerical model was proposed, based on the Arrhenius equation, to provide an insight on the morphological alterations during the switching process. This model provides an accurate quantitative description of the resistive switching mechanism with respect to the performed experimental data in this study.

## Methods

### Device Fabrication

Extraction of silk fibroin protein used for this investigation were carried out as previously described by Kaplan *et al* ^51^. Initially, Bombyx Mori cocoon pieces were boiled in 0.02 M Na_2_CO_3_ for 30 minutes and rinsed thoroughly with DI water as a degumming process to remove the Sericin protein binding the fibroin fibres. The extracted silk fibroin fibres were then dissolved in 9.3 M Lithium Bromide (LiBr) solution at 60 °C for 4 h. Subsequently, the obtained solution was dialyzed in DI water using the dialysis membrane with a cut-off molecular weight of 3.5 kDa for 72 h to remove LiBr impurities followed by centrifugation. The resultant purified silk fibroin solution is freeze dried and preserved for future usage under −40 °C refrigeration. The freeze-dried pellets were reconstituted to form 5 wt% silk fibroin solutions and filtered with a 0.2 µm syringe filter. The crossbar memristive device was fabricated on both a flexible biodegradable PVA substrate as well as a glass substrate. A layer of 10 nm Cr and 50 nm Au or 50 nm Pt were evaporated on a glass substrate using a Thermionics VE180 electron-beam evaporator at a pressure of 10^−7^ Torr. The corresponding gold-coated glass substrate was cleaned with isopropyl alcohol followed by de-ionised water in an ultrasonic bath for 8 minutes. The glass substrate was then patterned using laser ablation lithography (SUSS SLP300) to form the bottom electrodes. The reconstituted silk fibroin solution was spin-coated onto the patterned substrate at 1000 rpm for 1 min followed by water-annealing. The water annealing process involves the material being exposed to a high humidity environment. This was done by storing the device in a water chamber at −25 kPa for a period of 24 hrs. Cross-sectional imaging in Fig. S1 has shown that the spin-coated silk fibroin layer has a thickness of approximately 250 nm. To finalise the device, a 50 nm top electrode (Au, Cr or Ag) was evaporated onto the fibroin layer with the aid of a shadow mask. The bio-resorbable memristive device was initially fabricated by drop-casting PVA on a Teflon substrate followed by the spin-coating of PMMA A2 (2% PMMA/98% Anisole) at 1000 rpm for 1 min. Similar as mentioned above, a layer of 10 nm Cr and 50 nm Au or 50 nm Pt were evaporated. Next, the reconstituted silk fibroin solution was spin-coated onto the pattern substrate followed by water-annealing in a water chamber. The 50 nm Au top electrode was then e-beam evaporated through a shadow mask. To form the free-standing memristive device, the PVA substrate was lifted off the Teflon supporting structure via mechanical agitation.

### Cytotoxicity assessment

LDH assay: The LDH assay was performed using the Promega cytotoxic detection kit in conjunction with the Molecular Device SpectraMax M3. A 24 well culture plate was seeded with SH-SY5Y at a concentration of 10^3^ cells/ml. Test wells were prepared with dissolved sections of the memristor together with positive and negative controls as recommended by the LDH assay manufacturer. We tested different incubation times of the cells with the dissolved section (1, 24, 168 hrs) to verify the absence of chronic and acute toxicity. At different time point, the detection of LDH activity was performed by transferring 50 µl of culture solution to a 96 well plate together with the 50 µl of reaction solution. After 30 minutes, the stop solution was added and 490 nm wavelength adsorption was measured.

### Electrical Measurement

The electrical characterisation for the crossbar memristive devices were performed using the Agilent E5270B Precision IV Analyser in conjunction with a Cascade Microtech Summit Semi-Automated Probe Station and an ERS AirCool thermal regulation system. Chrono-amperometry was measured on the Cascade Microtech Summit Semi-Automated Probe with the chuck temperature set to 25 °C, 80 °C, 120 °C and 160 °C with different stimulation voltage supplied via the Agilent IV Analysers. Electrochemical measurement of the cyclic voltammograms (CV), were conducted on an electrochemical workstation at room temperature. CV measurements were carried out with a triangular voltage signal between −3 V and 3 V for Au-Pt and Au-Cu electrodes and between −1 V and 1 V for the Au-Ag electrode at scan rates of 50 mVs^−1^.

### Characterisation

FTIR-ATR spectrum measurements were obtained using the PerkinElmer FT-IR spectrometer over the 650 to 4000 cm^−1^ spectra with a resolution of 1 cm^−1^. AFM was used for the surface morphology and surface roughness analysis of silk fibroin films. AFM images were obtained with Agilent System (Agilent 5500 atomic force microscope) by using a commercially available silicon nitride cantilever with a force constant of 0.08 N m^−1^.

## Electronic supplementary material

Supplementary Information
