# Supplementary material for: A Silk Fibroin Bio-Transient Solution Processable Memristor
Source: Sci Rep. 2017 Nov 7;7:14731. doi: 10.1038/s41598-017-15395-5 (PMC5676789; doi:10.1038/s41598-017-15395-5)
Supplement: Supplementary file 1 — Supplementary Information [file 41598_2017_15395_MOESM1_ESM.doc]

Supporting Information for

A Silk Fibroin Bio-Transient Solution Processable Memristor

Jason Yong*^1,2,6^, Basem Hassan^1,2,6^, You Liang^1,2,6^, Kumaravelu Ganesan^3^, Ranjith Rajasekharan^2^, Robin Evans^2^, Gary Egan^6,7^, Omid Kavehei^4^, Jingliang Li^5^, Gursharan Chana^1,8,9,10^, Babak Nasr^1,2,6^, Efstratios Skafidas*^1,2,6^

^1^ Centre for Neural Engineering, The University of Melbourne, Carlton, VIC 3053, Australia.^2^ Department of Electrical and Electronic Engineering, University of Melbourne, VIC 3010, Australia.^3^ School of Physics, University of Melbourne, Melbourne, VIC 3010, Australia.^4^ School of Engineering, RMIT University, Melbourne, VIC 3001, Australia.^5^ Institute for Frontier Materials, Deakin University, Geelong, VIC 3216, Australia.^6^ Australian Research Council Centre of Excellence for Integrative Brain Function, The University of Melbourne, Victoria, 3010, Australia. ^7^ Monash Biomedical Imaging, Monash University, Clayton, VIC, Australia.^8^ Department of Medicine (RMH), The University of Melbourne, VIC, 3010, Australia.^9^ Department of Psychiatry, The University of Melbourne, VIC, 3010, Australia.^10^ Florey Institute of Neuroscience and Mental Health, Parkville, VIC 3010. Correspondence and requests for materials should be addressed to Jason Yong. (email: jasony1@student.unimelb.edu.au)


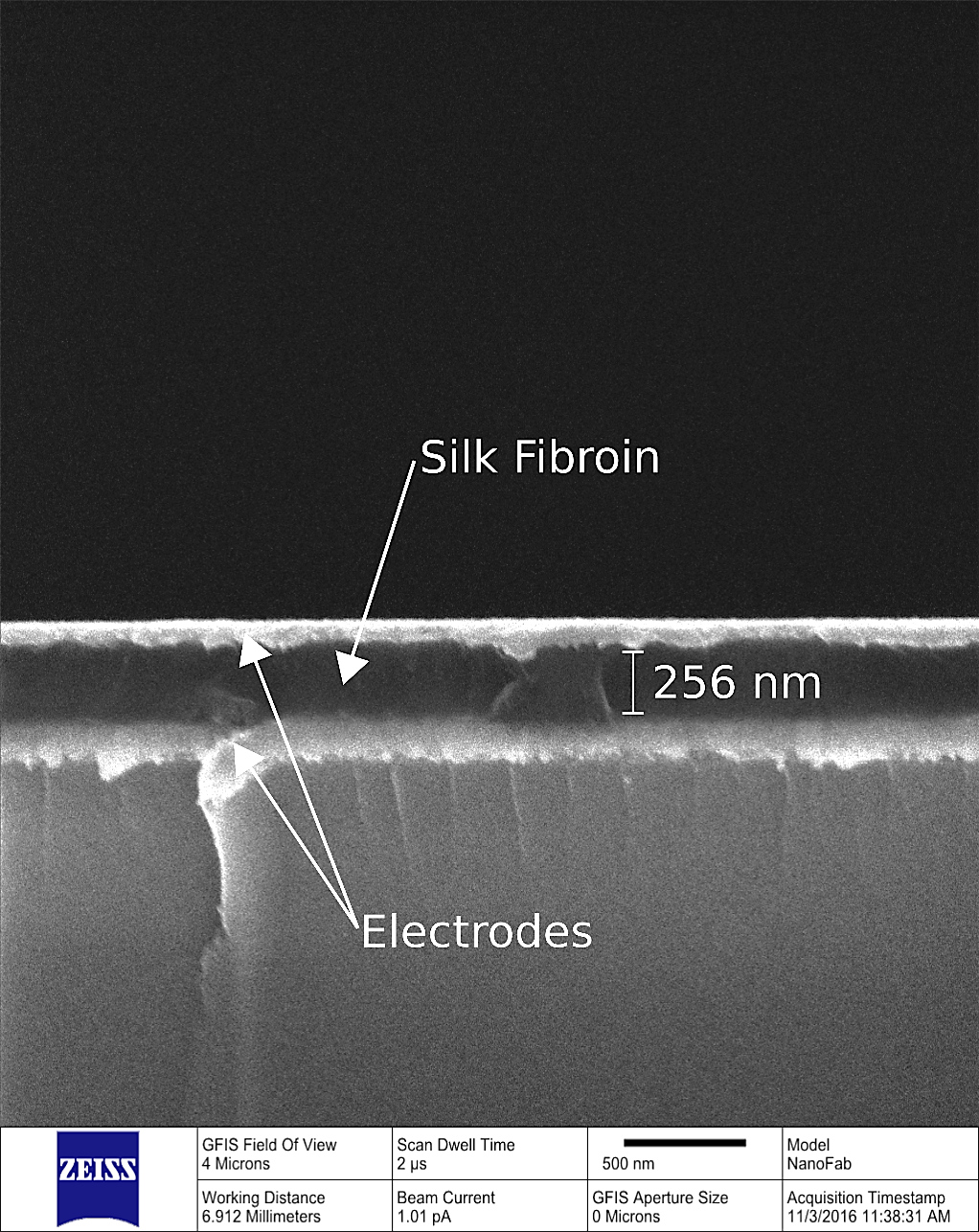


Figure S1. Helium ion microscopy (HIM) images showing the cross-sectional image on the silk fibroin film interposed between metal electrodes. Imaging was performed via the Helium Ion Microscope, HIM (Carl Zeiss, Orion Nanofab, Peabody MA, USA) operating at an accelerating voltage of 30 and a beam current of approximately 1 pA.


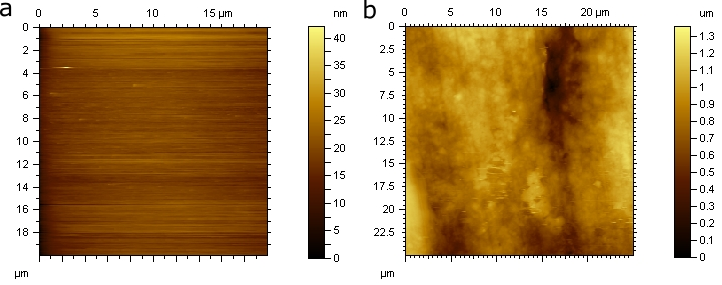


Figure S2 Surface topography of the silk fibroin film on a) glass substrate and b) PVA substrate. The silk fibroin film produced on the PVA substrate shows significantly larger surface roughness as compared to the silk fibroin film deposited on the glass substrate. The larger surface roughness originates from the Teflon substrate used to ease the lift of process of the silk fibroin film. Imaging were performed using the Agilent 5500 atomic force microscope.


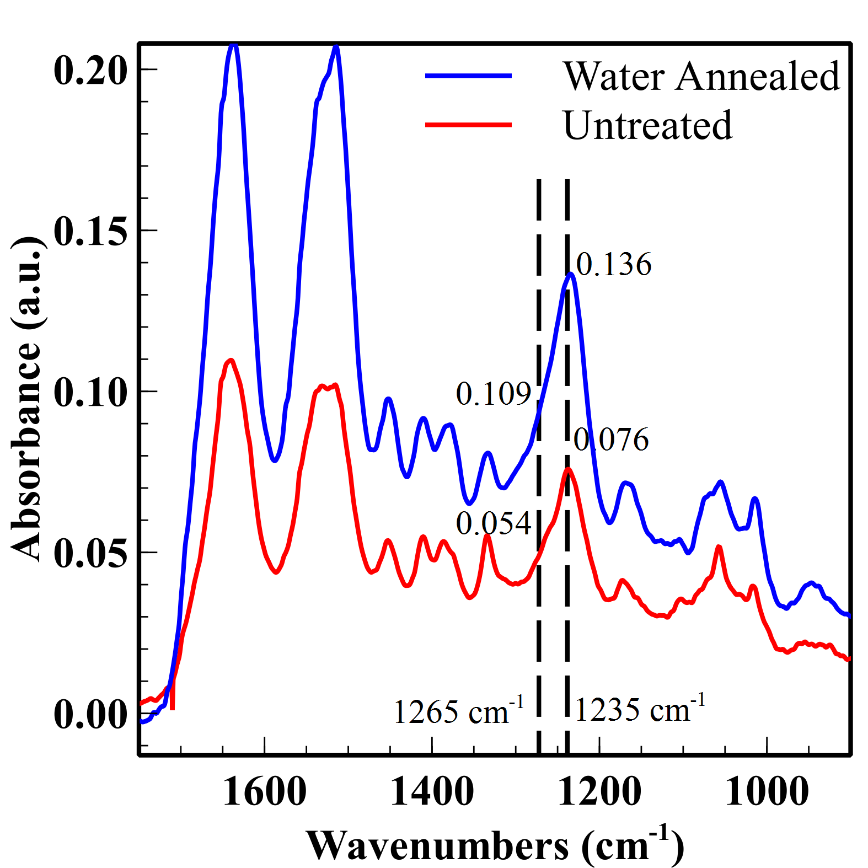


Figure S3. FTIR-ATR spectrum of the water annealed and untreated silk fibroin film. It shows the absorption strength ratio of the spectrum 1265 cm^-1^ and 1235 cm^-1^ (A_1265_ / A_1235_) which is commonly used to determine the crystallinity index. The water annealed silk fibroin films obtained a higher crystallinity index of 0.80 whereas the untreated films had an index of 0.71.


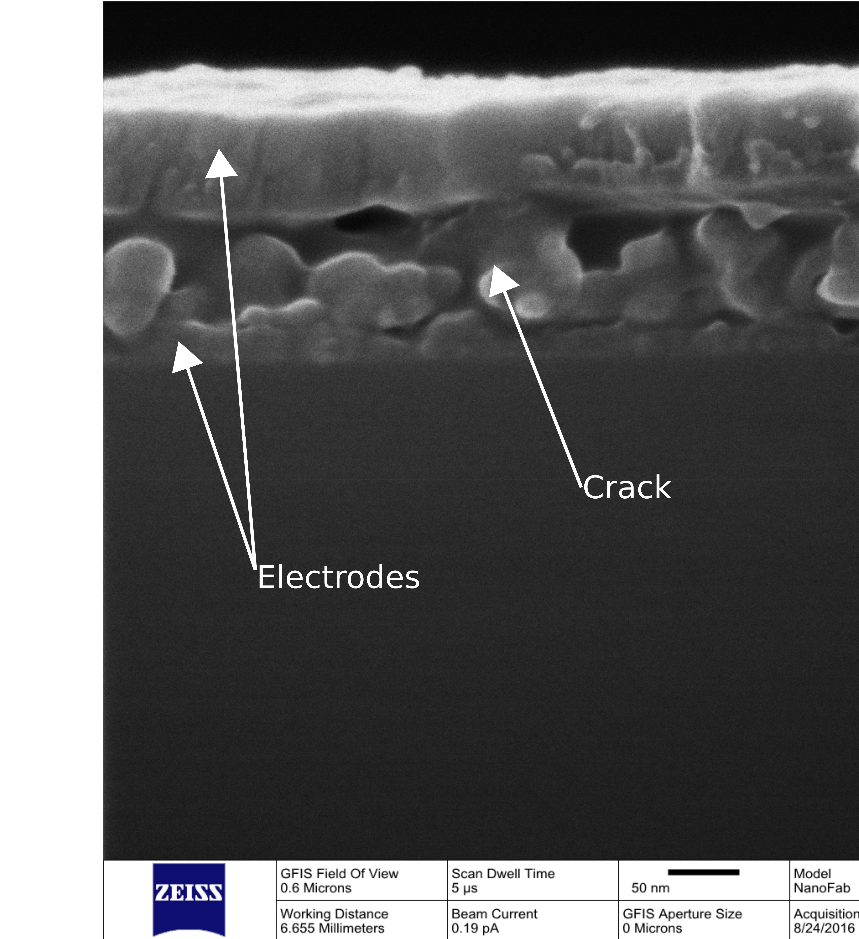


Figure S4. Helium ion microscopy (HIM) images showing formations of cracks in untreated silk fibroin film sandwiched between metal electrodes. The bright contrast between the metal electrodes indicates the penetration defects within the silk fibroin silk during the evaporation process. Imaging was performed via the Helium Ion Microscope, HIM (Carl Zeiss, Orion Nanofab, Peabody MA, USA) operating at an accelerating voltage of 30 and a beam current of approximately 0.19 pA. Surface charge compensation achieved with support of the electron flood gun.


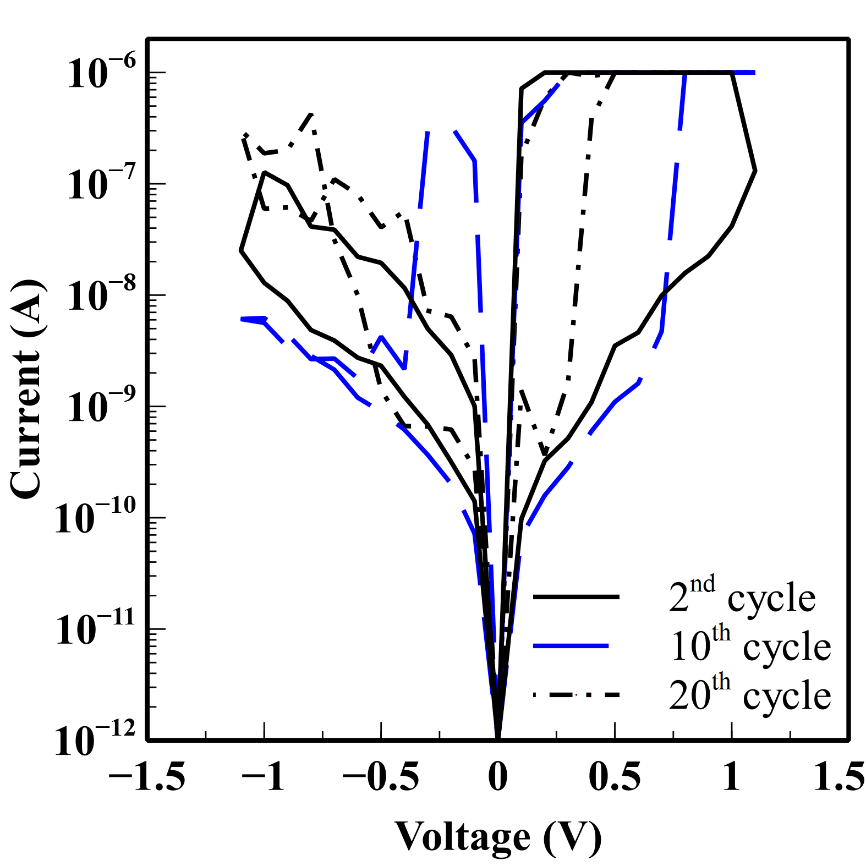


Figure S5. IV characteristics for an Au-Silk Fibroin-Ag memristor showing the earlier and subsequent cycles. The subsequent read-write cycles show less distinctive off and on resistance state as the memristive device approaches the endurance limit.


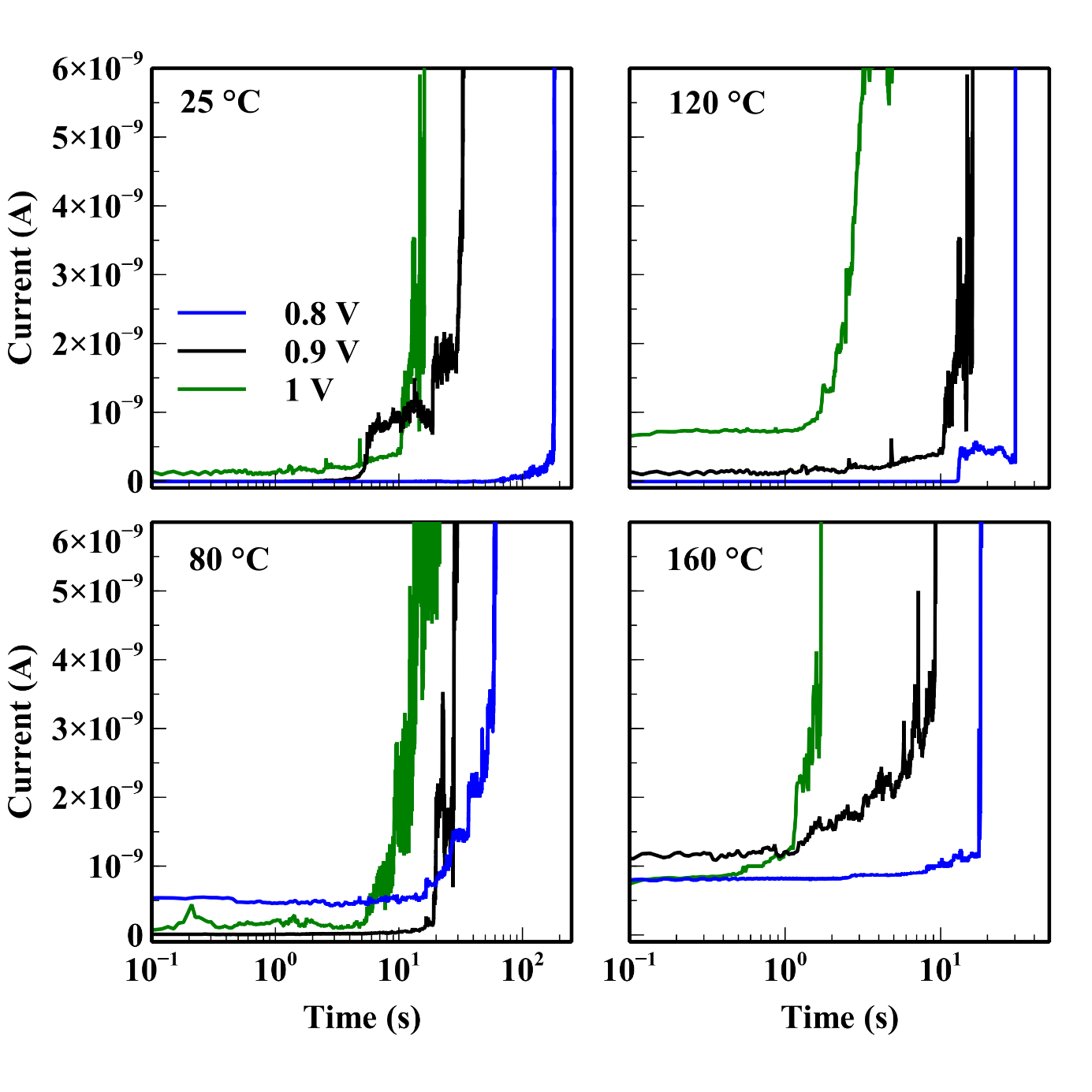


Figure S6. Chrono-amperometry for Au-Ag electrodes with a bias voltage of 0.8 V to 1 V. The blue, black and green solid line represents a bias voltage of 0.8V, 0.9V and 1.0V respectively. These chrono-amperometry measurements were performed with an ambient temperature of 25 °C, 80 °C, 120 °C and 160 °C.


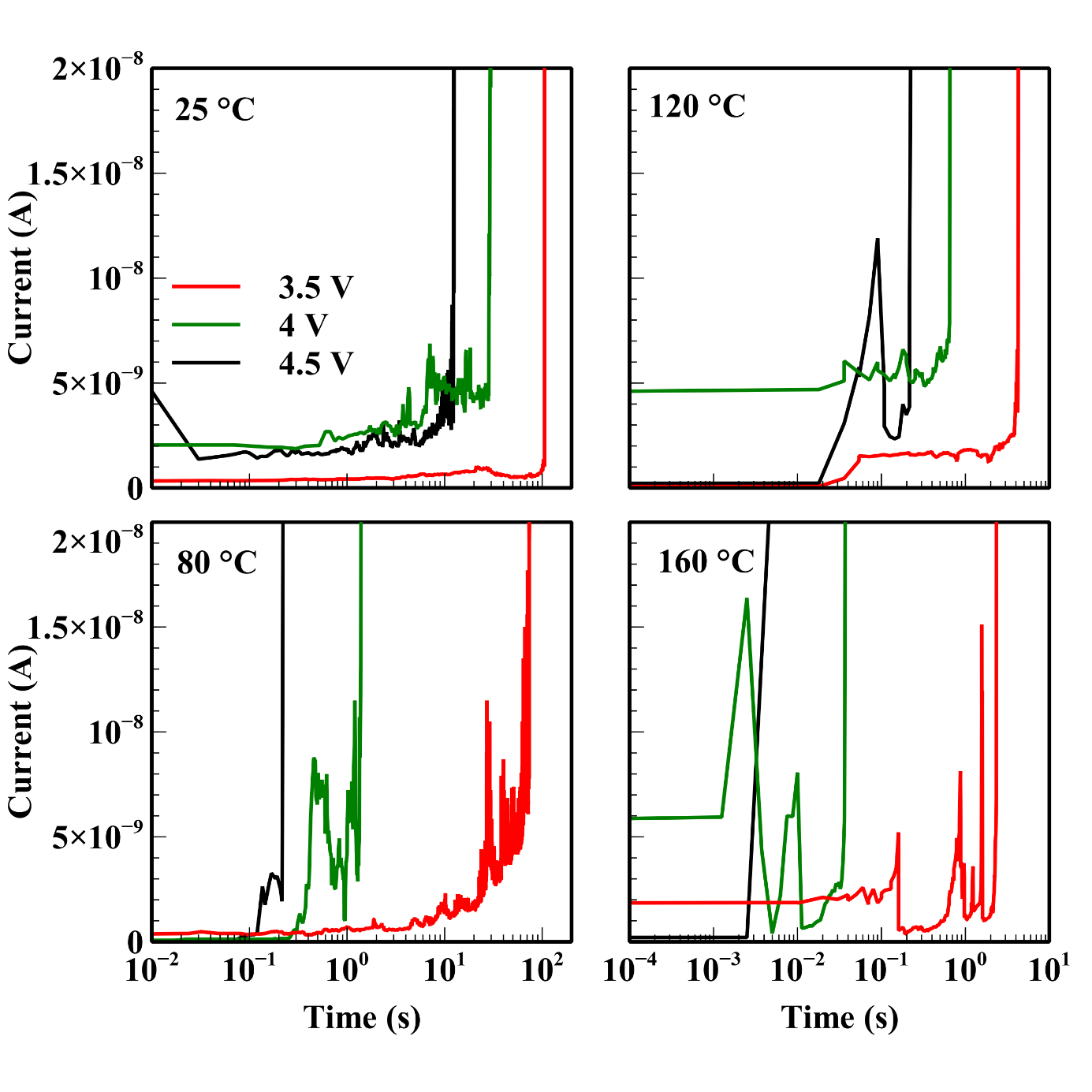


Figure S7. Chrono-amperometry for Au-Cu electrodes with a bias voltage of 3.5 V to 4.5 V. The red, green and black solid line represents a bias voltage of 3.5V, 4.0V and 4.5V respectively. These chrono-amperometry measurements were performed with an ambient temperature of 25 °C, 80 °C, 120 °C and 160 °C.


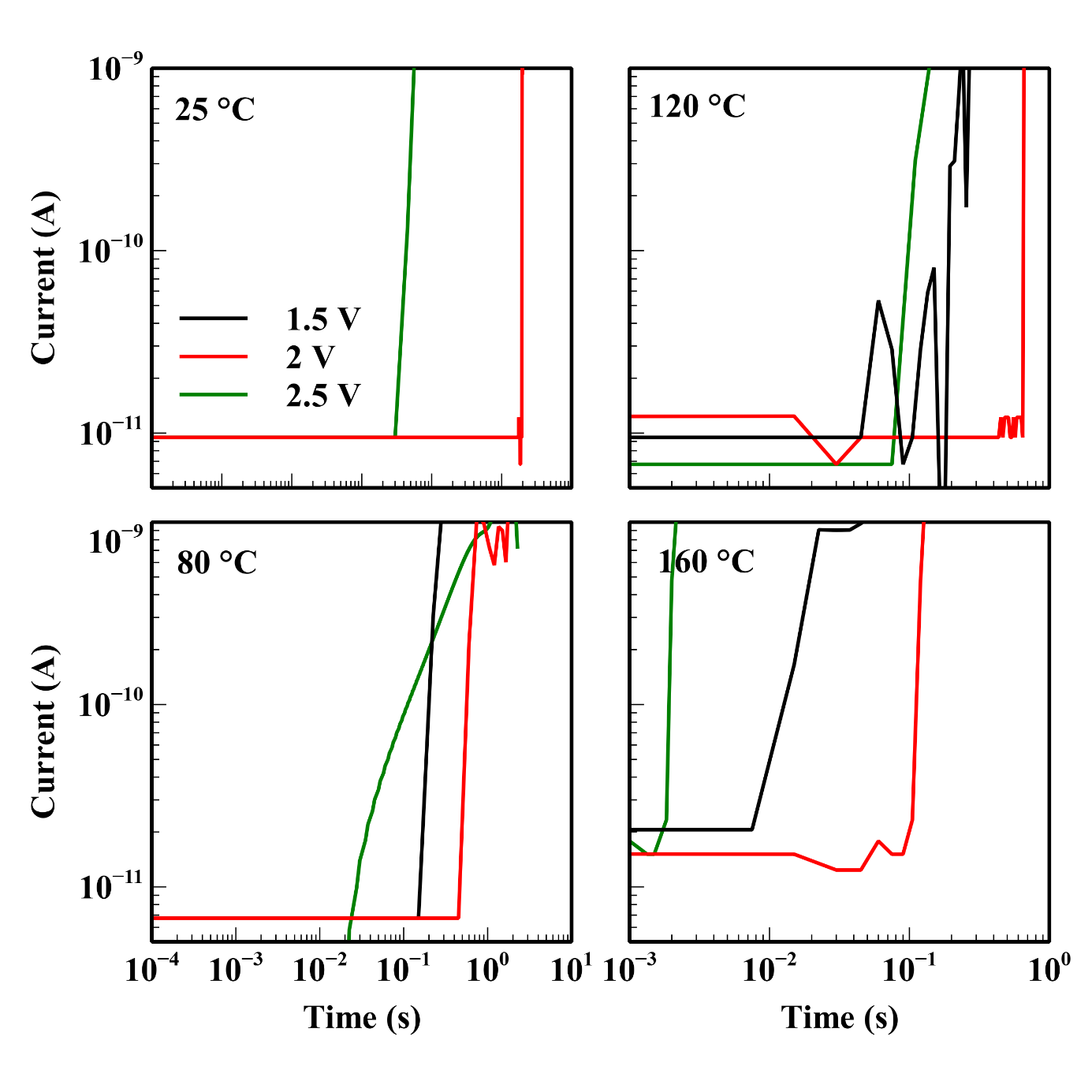


Figure S8. Chrono-amperometry for Au-Pt electrodes with a bias voltage of 1.5 V to 2.5 V. The red, green and black solid line represents a bias voltage of 1.5V, 2.0V and 2.5V respectively. These chrono-amperometry measurements were performed with an ambient temperature of 25 °C, 80 °C, 120 °C and 160 °C.


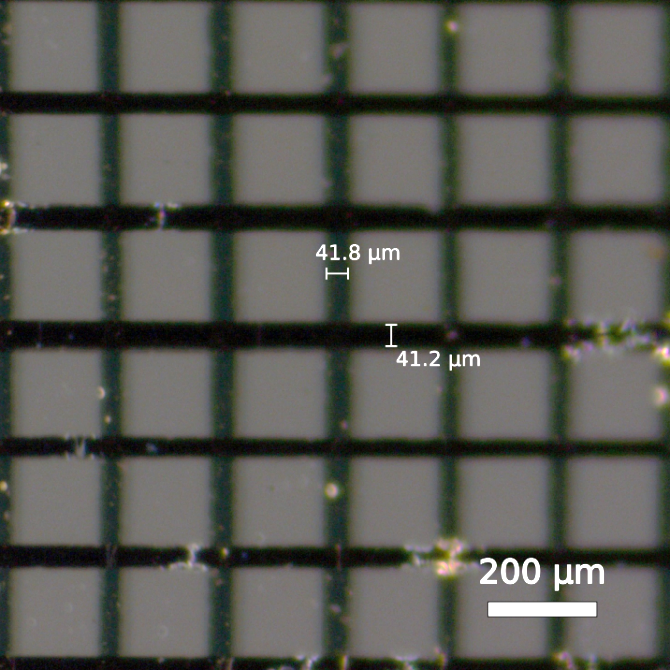


Figure S9. Optical image showing the dimension of the crossbar memristive device.


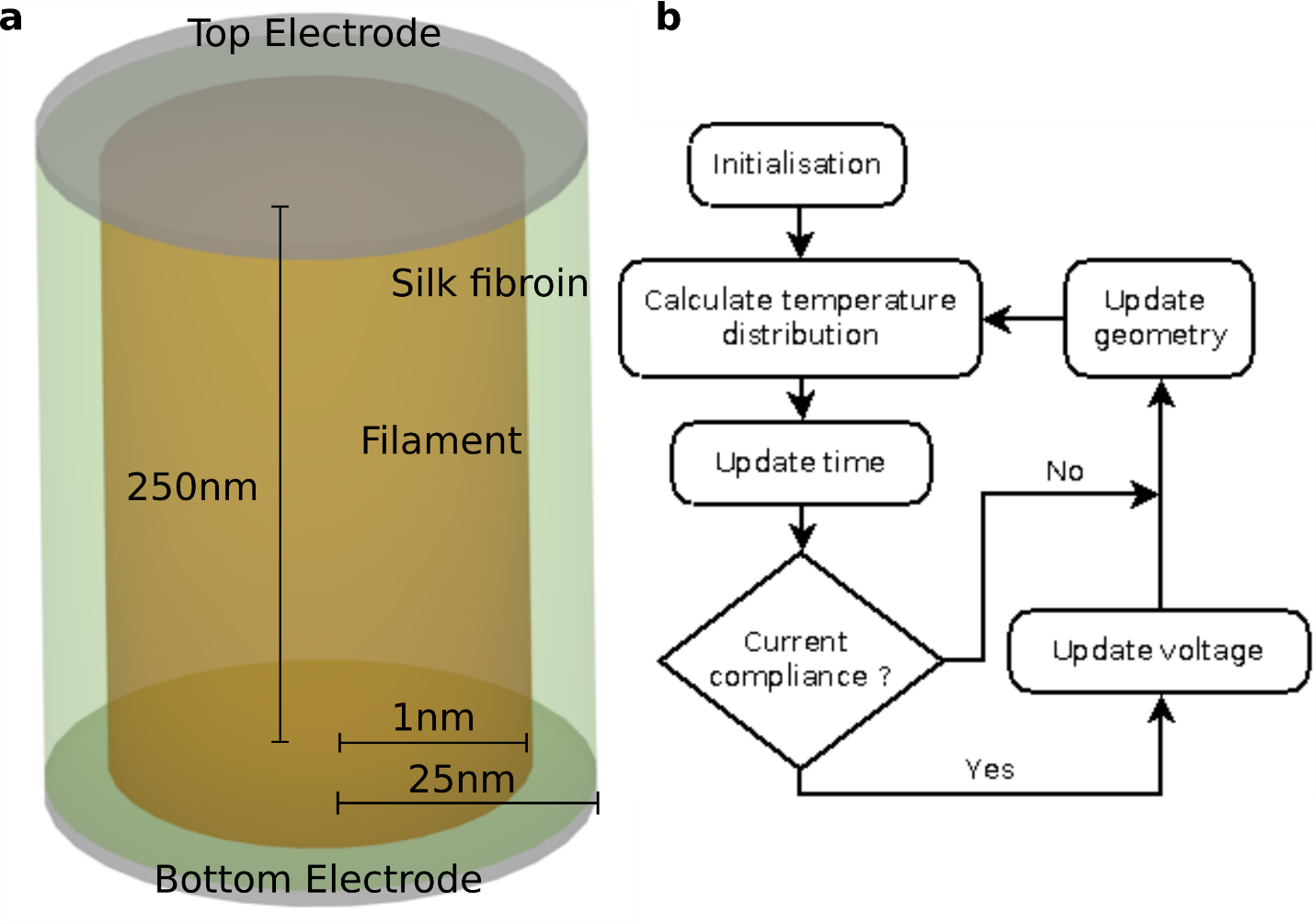


Figure S10. Simulation geometry definition and process flow. (a) Simulated geometry used in the numerical solver. (b) Simulation flowchart.


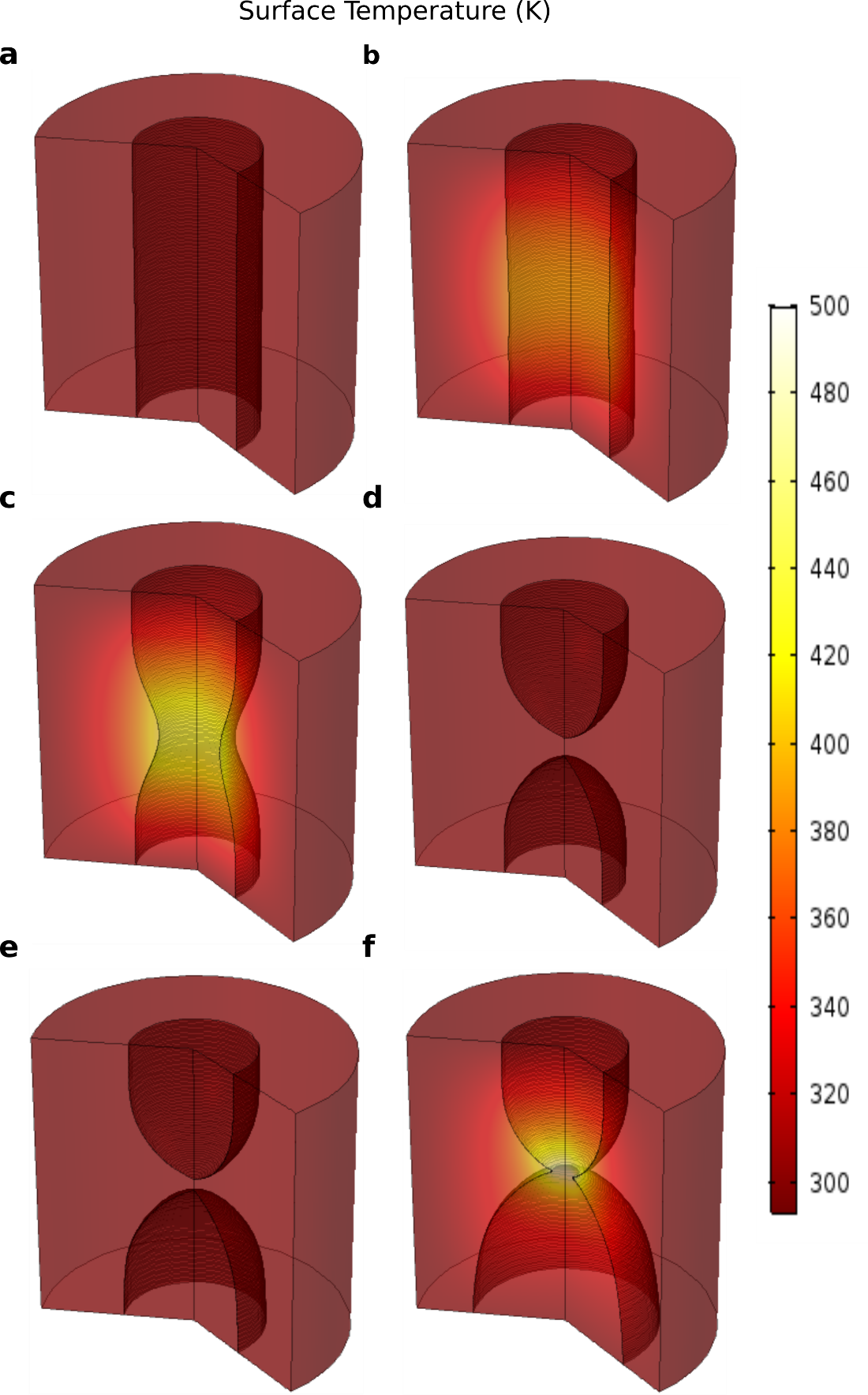


Figure S11. Surface plot for the temperature distribution and morphology of the conductive filament. The illustration shows the sequential cycle of the SET/RESET process for an applied voltage of (a) 0V, (b) -0.2V, (c) -0.3V, (d) -0.5V, (e) 0.5V, (f) 0.7V using parameters for the Au-Ag electrodes.

Table S1. Electrode combination for silk fibroin memristors and their proposed switching mechanism.

| Electrode material combination | Proposed Switching mechanism | Ref |
| --- | --- | --- |
| Al-fibroin-ITO | Oxidation and reduction of silk fibroin protein chain | 6 |
| Mg-fibroin-Mg | Electrochemical metallization | 18 |
| Al-fibroin-ITO | Oxidation and reduction of silk fibroin protein chain | 14 |
| Ag-fibroin-Au | Electrochemical metallization | 57 |

Table S2. Numerical simulation fitting parameters. The following fitting parameters are used in the simulation model to replicate the experimental data.

| Fitting Parameters | Electrode Combinations | | |
| --- | --- | --- | --- |
|  | Ag-Au | Cu-Au | Au-Pt |
| A_set_ (m/s) | 5×10^-6^ | 5×10^-6^ | 5×10^-6^ |
| A_reset_ (m/s) | 5×10^-6^ | 5×10^-6^ | 5×10^-6^ |
| n | 4×10^-3^ | 8×10^-4^ | 5×10^-3^ |
